# Community-Based Control of Malaria Vectors Using *Bacillus thuringiensis* var. *Israelensis* (*Bti*) in Rwanda

**DOI:** 10.3390/ijerph19116699

**Published:** 2022-05-30

**Authors:** Emmanuel Hakizimana, Chantal Marie Ingabire, Alexis Rulisa, Fredrick Kateera, Bart van den Borne, Claude Mambo Muvunyi, Michele van Vugt, Leon Mutesa, Gebbiena M. Bron, Willem Takken, Constantianus J. M. Koenraadt

**Affiliations:** 1 Malaria and Other Parasitic Diseases Division, Rwanda Biomedical Centre (RBC), Ministry of Health, Kigali 7162, Rwanda; 2 Laboratory of Entomology, Wageningen University & Research, 6708 PB Wageningen, The Netherlands; willem.takken@wur.nl; 3 Research Department, Community-Based Socio-Therapy, Kigali 4560, Rwanda; cingabire7@gmail.com; 4 Department of Cultural Anthropology and Development Studies, Radboud University, 6525 GD Nijmegen, The Netherlands; alexis.rulisa@gmail.com; 5 Partners in Health, KG 9, Avenue 46, Kigali 3432, Rwanda; fkateera2011@gmail.com; 6 Department of Health Promotion, Maastricht University, 6200 MD Maastricht, The Netherlands; b.vdborne@maastrichtuniversity.nl; 7 Department of Clinical Biology, School of Medicine and Pharmacy, College of Medicine and Health Sciences, University of Rwanda, Kigali 3286, Rwanda; clmuvunyi@gmail.com; 8 Academic Medical Center, 1105 AZ Amsterdam, The Netherlands; m.vanvugt@amsterdamumc.nl; 9 Centre for Human Genetics, College of Medicine and Health Sciences, University of Rwanda, Kigali 4285, Rwanda; lmutesa@gmail.com; 10 Quantitative Veterinary Epidemiology, Wageningen University & Research, 6708 PB Wageningen, The Netherlands; bieneke.bron@wur.nl

**Keywords:** larval source management, vector control, *Bacillus thuringiensis* var. *israelensis*, community engagement

## Abstract

Larval source management (LSM) programs for control of malaria vectors are often vertically organized, while there is much potential for involving local communities in program implementation. To address this, we evaluated the entomological impact of community-based application of *Bacillus thuringiensis* var. *israelensis* (*Bti*) in a rice irrigation scheme in Ruhuha, Rwanda. A non-randomized trial with control compared a *Bti* implementation program that was supervised by the project team (ES) with a program that was led and carried out by local rice farming communities (CB). One other area served as a control to assess mosquito populations without *Bti* application. Entomological surveys were carried out every two weeks and assessed the presence and abundance of the larval, pupal, and adult stages of *Anopheles* mosquitoes. In ES, the per round reduction in *Anopheles* larval habitats was estimated at 49%. This reduction was less in CB (28%) and control (22%) although the per round reduction in CB was still significantly higher than in control. Pupal production was almost completely prevented from round 5 (out of 10) onwards in both CB (average habitat occupancy 0.43%) and ES intervention arms (average habitat occupancy 0.27%), whereas pupal occupancy rates were on average 12.8% from round 5 onwards in the control. Emergence of adult mosquitoes from rice fields was thus prevented although this was not directly noticeable in adult *An. gambiae* populations in houses nearby the rice fields. Together with our earlier work on the willingness to financially contribute to the LSM program and the high perceived safety and acceptance of the *Bti* product, the current study demonstrates that, in an environment with limited resources, communities could become more engaged in LSM program implementation and contribute directly to malaria vector control in their environment.

## 1. Introduction

The impact of long-lasting insecticide-treated nets (LLIN) and indoor residual spraying (IRS) in the global fight against malaria encouraged several malaria-endemic countries, including Rwanda, to set up the ambitious goal of malaria elimination [1,2,3]. At the same time, the occurrence of residual malaria transmission and local immigration of infected people present challenges to the ultimate goal of malaria elimination in highly endemic regions [4]. As residual malaria transmission can result from changes in the behavior of malaria vectors, malaria elimination may thus not be feasible without additional and innovative interventions [5].

Several studies demonstrated that mosquitoes have developed the ability to avoid contact with insecticide-treated surfaces [5,6]. Other examples of behavioral change include earlier biting when people are not yet protected by bed nets and trends of outdoor biting and resting of malaria vectors that were formerly active inside houses [7,8,9]. Other malaria vector species have developed the habit to bite domestic animals and, in this way, escape the risk of contact with insecticides [10,11]. Residual transmission can also result from a change in vector species composition, whereby secondary vectors acquire high transmission capacity and replace the primary vectors, mainly due to ecological and climatic changes [12,13,14]. Moreover, the scaling up of insecticide-based vector control interventions has contributed to the selection of resistant mosquito strains, which are not killed by the standard dose of the different types of insecticides. This insecticide resistance has been spreading at an alarming rate [15,16]. The resistance of malaria parasites to anti-malarial drugs is another hindrance to malaria control, which continuously requires adaptation and a search for new, cost-effective measures [17].

To tackle the challenge of insecticide resistance, alternative interventions are required that can be implemented in integrated vector management (IVM) programs as part of malaria control [18,19,20]. Larval control interventions have proven effective across a range of different settings and include environmental management, application of insect growth regulators, as well as chemical and biological control [21]. Several field trials using the biological larvicide *Bacillus thuringiensis* var. *israelensis* (*Bti*) have been carried out in Tanzania, Kenya, the Gambia, and Benin. These showed a substantial impact on key parameters of malaria transmission, such as a reduction of the occupancy rates and density of mosquito larval and pupal stages in aquatic habitats, the risk of human biting by mosquitoes, the entomological inoculation rate, and the prevalence or incidence of malaria [22,23,24,25,26].

A key question that remains, however, is whether such approaches can be implemented by local communities themselves rather than through a vertically organized malaria control program. These communities could constitute ever-present and knowledgeable drivers of the intervention. Our earlier research with rice-farming communities in Rwanda on the socio-economic perspectives of larval source management (LSM) demonstrated that communities perceived *Bti* as safe, which increased the likelihood of communities participating in the program through investment in labor time for application of *Bti* [27]. It was estimated that these rice-farming communities were willing to contribute 15–25% of co-financing to the program [28]. In this study, implemented in the framework of the Rwanda IVM strategy, we evaluated the entomological impact of community-based application of *Bti* in a rice irrigation scheme.

## 2. Materials and Methods

### 2.1. Study Site

**Figure 1:**
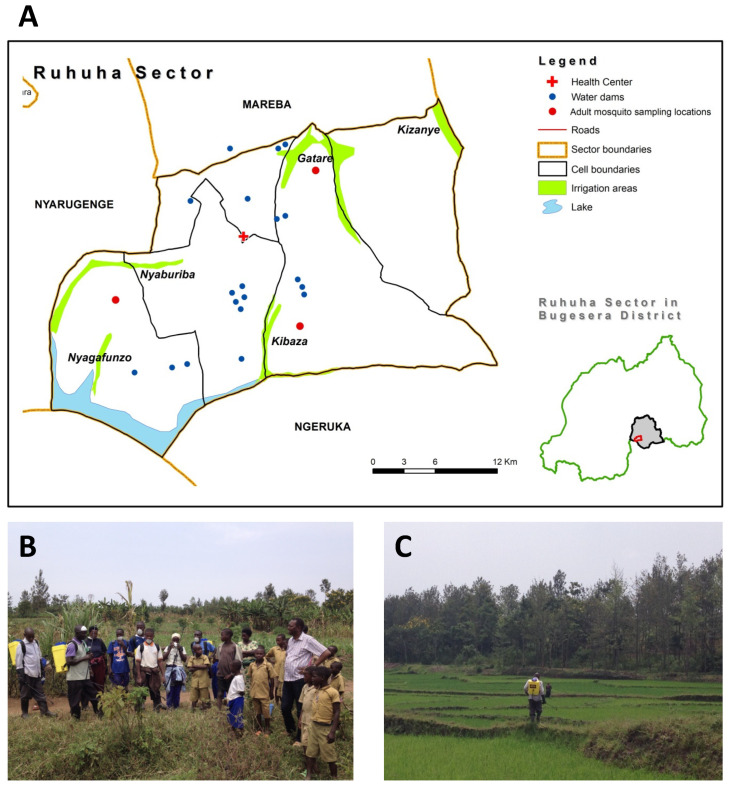
(**A**) Location of the experimental sites in Ruhuha, southeast Rwanda. Nyaburiba: rice field control, Gatare: rice field-1 for community-based application of *Bti* (CB1), Kizanye: rice field-2 for community-based application of *Bti* (CB2), Kibaza: rice field-1 for expert supervised application of *Bti* (ES1), Nyagafunzo: crop land-2 for expert supervised application of *Bti* (ES2). Blue dots represent the 19 water hill dams at which expert-supervised application of *Bti* (ES3) was also carried out. (**B**) Training of community representatives for spraying of *Bti*. (**C**) Spraying of *Bti* using knapsack sprayers in the irrigated rice fields.

The study was carried out in Ruhuha, one of the fifteen sectors of Bugesera district in south-eastern Rwanda (Figure 1). The area is located 42 km south of the capital Kigali. The sector is divided into five administrative cells and 35 villages. The elevation ranges from 1300 to 1573 m above sea level. It has an estimated population of 24,000 people and over 5000 households. The sector has one health center with a network of 140 community health workers (CHWs) and 105 members of community malaria actions teams (CMATs) [29,30], who support implementation of health activities in the community. The area is drained by five marshlands, and rice is grown in four irrigated rice fields covering an estimated area of 93 ha: Kibaza (27 ha), Gatare (25 ha), Nyaburiba (33 ha), and Kizanye (8 ha). The remaining marshland of Nyagafunzo (8 ha) is used to grow subsistence crops. The major malaria prevention strategies implemented are long-lasting insecticide-treated nets (LLINs) and two annual rounds of indoor residual spraying (IRS) using non-pyrethroid insecticides.

### 2.2. Treatment Arms for Larval Source Management Using Bti

According to WHO guidelines, our intervention study is classified as a “non-randomized trial with control” [31] and included one control and two larval source management arms. Baseline information on potential mosquito breeding sites was collected in the study area as well as on stakeholder engagement and socio-economic status. All permanent or semi-permanent water bodies were mapped prior to the intervention and later assigned to a treatment arm (Figure 1, Appendix A). These included the four marshlands used for irrigated rice growing (Nyaburiba, Kibaza, Gatare, and Kizanye), one marshland used by the community for growing seasonal subsistence crops (Nyagafunzo), and 19 so-called hill dams for harvesting rain water (each approximately 200 m^2^ in size). Nyaburiba (35 ha) was selected as the control arm, without any application of *Bti*. For the “expert-supervised” arm (hereafter referred to as ES), Kibaza (27 ha; entitled ES1), Nyagafunzo (8 ha; ES2), and all 19 water hill dams (ES3) were selected. For the third arm, “community-based” application of *Bti* (hereafter referred to as CB), Gatare (25 ha, CB1) and Kizanye (8 ha; CB2) marshlands were selected. The minimum distance between intervention arms was estimated at 5 km.

One month before the *Bti* application, a baseline survey on the perception, engagement, and willingness to pay for LSM was carried out by a team consisting of a sociologist and an economist [27,28]. At the same time, the entomological sampling sites were identified, numbered, and mapped. This activity was followed by baseline entomological surveys on larval and adult stages of mosquitoes and led by trained entomology technicians in collaboration with community members.

### 2.3. The Selection of Implementers for Bti Application

The 39 community members recruited for application of *Bti* were selected in collaboration with the four local cooperatives of rice farmers and CMATs. Of them, 11 (28%) were CMAT members, and 28 (72%) were rice farmers. Initially, the heads of rice-farmer cooperatives and representatives of CMATs participated in the process to define the selection criteria for sprayers of *Bti* as well as entomology monitoring surveyors. Two criteria were mutually agreed upon: (1) sprayers should be members of CMATs and/or be a rice farmer as well as inhabit one of the villages neighboring the areas targeted for treatment with *Bti*, and (2) surveyors for monitoring of larval and adult mosquitoes should only be selected from the members of CMATs.

For the CB intervention arm, a team of 20 sprayers was selected by the heads of cooperatives of rice farmers and approved by the research team. Another team of 19 sprayers for the ES intervention was recruited by the research team through a semi-structured interview. In addition to the sprayers, two independent teams of community members were deployed for larval and for adult mosquito monitoring of the intervention, respectively. These were separately recruited by the research team among the members of CMATs.

### 2.4. Organizational Structures of Intervention Arms

The major distinction between the two intervention arms (ES and CB) was based on the organization and degree of supervision, the logistical management of *Bti,* and the frequency of reporting (Table A1). For ES, the supervision of sprayers and the logistical management was carried out by members of the research team (author E.H.). The reporting was done daily to the office of the research team established at the Ruhuha health center, located centrally in the study area. For the CB arm, the application of *Bti* was entirely the responsibility of the cooperatives of rice farmers. The head of the cooperatives ensured supervision of the spraying with an overall management of logistics (distribution of spray pumps, the *Bti* product, etc.) and reported weekly to the research team. The research team did not interfere in this arm and deliberately sought to find out potential hurdles (logistical, financial, and social) for community-based LSM.

For the CB arm, the *Bti*, spray pumps, personal protective equipment, and reporting forms were stored free of charge at the office of the rice cooperative, while all *Bti* spraying commodities and reporting material used in the ES arm were stored and managed at the Ruhuha health center.

Spraying took place every week from 7:00 a.m. until 1:00 p.m. in the ES arm. Working times for coordinators of the CB team ranged from 7:00 a.m. to 2:00 p.m. The monitoring of larval and adult mosquitoes was conducted by independent teams selected from CMAT members and supervised by a trained entomology technician for each marshland that was part of the intervention.

### 2.5. Bacillus thuringiensis var. Israelensis as Biological Larvicide

The larvicide applied for LSM was *Bacillus thuringiensis* var. *israelensis*, strain AM 65-52 (*Bti*), commercially traded under the name of VectoBac^®^ Water-Dispersible Granules (WDG), 3000 International Toxic Units (ITU) per mg. The product was supplied by Valent Biosciences Corporation. The active ingredient of the product is based on a mixture of free endotoxin protein crystals produced by *Bti* strain AM65-52 and the spores and cells bearing them [32]. The average half-life of the product in water bodies in the field is estimated to be one week [33].

### 2.6. Training and Calibration of Equipment

A five-day training of sprayers for application of *Bti* was supported by Valent Biosciences Corporation (Valent Biosciences, Libertyville, IL, USA). It covered the techniques of application of *Bti*, including the calibration of sprayer pump nozzles, the spraying speed, flow and application rates, as well as the reporting procedures and schedule. A simulation of spraying using water was conducted in the field after the theoretical training sessions.

The application was calibrated at a spraying speed of 50 m per minute, a swath width of 8 m, a flow rate of 1.2 L/min, and an application rate of 30 L of *Bti* suspension per hectare. As the application dosage recommended by the manufacturer was 300 g of *Bti* per ha (0.3 kg/ha), this quantity was obtained by mixing three times 100 g of the granules in 10 L of water in the sprayer tank.

### 2.7. Entomological Surveys

**Figure 2:**
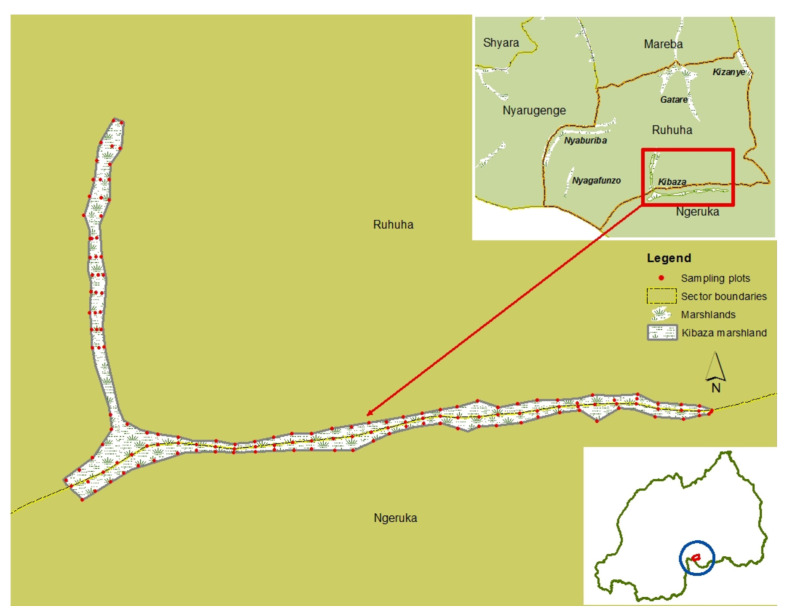
Example of sampling sites for mosquito larval stages in the Kibaza rice field-1 (ES1), Ruhuha, Rwanda.

Surveys for larval and adult stages of mosquitoes were carried out for two consecutive days/nights one month before the application of *Bti* (baseline) as well as every two weeks for six consecutive months, from February to July 2015, covering one cycle of rice cultivation. The individual farming plots of 20 m × 20 m for larval sampling per marshland were marked using a Global Position System (GPS) device at every 100 m and following three transects, respectively, along the central irrigation channel and the two edges of rice fields (Figure 2, Table 1). The geo-referenced plots were then numbered, coded, digitized, and mapped using Geographic Information System software (ArcGIS Desktop 10.8, version 10.7.0.10450, ESRI, Redlands, CA, USA). The owner’s name for each sampling plot was also recorded for easier identification by the local surveyors. At each intervention site, the representative rice farmer, who later participated in larval monitoring, contributed to the geo-coding of sampling plots and identification of the respective owners.

The aquatic stages were collected with standard dippers (350 mL) with 10 dips per surveyed plot of 20 m × 20 m [34]. Throughout the surveys, early instars (L1 + L2), late instars (L3 + L4), and pupae were separately recorded on a sampling form. At each round and one to two days post *Bti* application, the larval survey was conducted for two consecutive days. The density of late instar *Anopheles* larvae and habitat occupancy for *Anopheles* larvae and pupae were calculated.

Adult mosquitoes were sampled every two weeks for two consecutive nights using miniature CDC light traps (Model 512; John W. Hock Company, Gainesville, FL, USA) suspended one meter above the floor and at the foot end of the bed where an occupant was sleeping [35]. In one village bordering the control and each *Bti* intervention arm, 10 houses were purposively selected for collection of adult mosquitoes. The traps were set up at 6:00 p.m. and retrieved the next morning at 6:00 a.m. by the trained members of CMATs. At the laboratory, mosquitoes were first identified to species level using standard morphological identification keys [36]. The sibling species were identified using a polymerase chain reaction (PCR) assay [37] from a sample of the total *An. gambiae* s.l. collected.

### 2.8. Data Analysis

To evaluate the impact of the intervention on larval and pupal development in the rice fields and open-water breeding habitats, we constructed generalized linear mixed models (GLMM). We hypothesized that presence of the aquatic stages would decline over the rice cycle, as continued rice growth increasingly hampers oviposition even in the absence of any intervention. However, we expected that this decline would be stronger in the two treatment arms compared to the control arm. Hence, we were specifically interested in the significance of the interaction term between intervention and the round of sampling. In the model, sampling site was included as a random variable, nested within one of the five marshland areas. Occupancy was modeled using a binomial distribution. The catches of adult mosquitoes were fitted to a negative binomial distribution with log link function and were analyzed using GLMM, with house included as a random variable. Statistical analysis was conducted using RStudio software (version 1.2.1335), with the *lme4* package for model construction, *ggplot2* for data visualization, and *sjPlot* for plotting statistical model predictions.

## 3. Results

### 3.1. Impact on Anopheles Larval Stages

**Figure 3:**
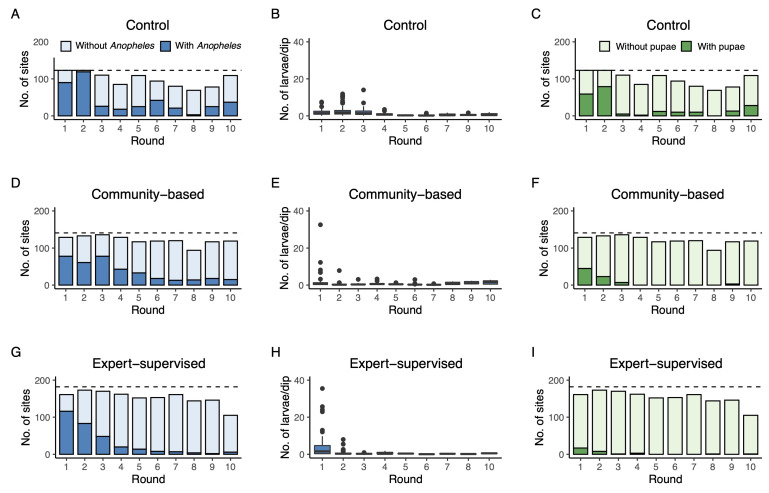
Number of sites with and without *Anopheles* larvae per survey round in the control arm (panel (**A**)), number of *Anopheles* larvae per dip per survey round in the control arm (panel (**B**)), and number of sites with pupae per survey round in the control arm (panel (**C**)). Similarly, in panels (**D**–**I**), the same outcome parameters are shown for the CB arm and ES arm, respectively. The dashed horizontal lines represent the maximum number of sampling sites that were selected per intervention arm (see Table 1).

**Figure 4:**
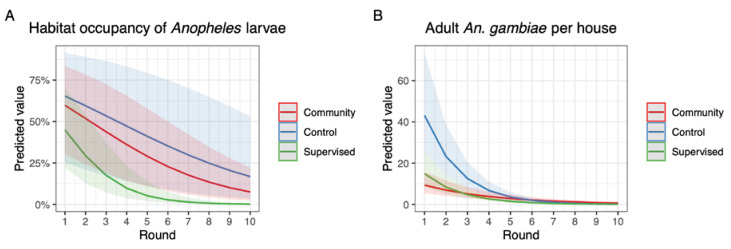
Predicted habitat occupancy rates for *Anopheles* larvae over the survey rounds (panel (**A**)). Predicted numbers of adult *An. gambiae* per house per survey round (panel (**B**)).

The habitat occupancy of sampling sites with *Anopheles* larvae was high for all three arms at the baseline (round 1: 60% in CB, 72% in ES, and 73% in control, Figure 3A,D,G). In subsequent survey rounds, these proportions dropped substantially in ES and CB to 6 and 13%, respectively, in the final round (round 10). In the control arm, occupancy rates also dropped over time but remained more variable. In round 10, the occupancy rate was still 34%. A similar decline over time was seen in the actual numbers of *Anopheles* larvae per dip in those sites that did contain larvae (Figure 3B,E,H). Most strikingly, larval densities rapidly decreased to very low numbers after round 1 in CB and ES, whereas densities increased in the two subsequent rounds in the control. The GLMM of the habitat occupancy rate with sampling site included as a random variable showed a significant interaction between treatment and round, suggesting that declines in habitat occupancy rate were different among the control, CB, and ES arms (Table 2). Based on the parameter estimates, the per round reduction for the ES arm was calculated at 49.0%, which was significantly higher than the estimated per round reduction in control (22.0%; *z*-value = −8.53, *p* < 0.001) and CB arms (27.6%). Furthermore, the reduction in CB was significantly higher than in control although less strong (*z*-value = −2.00, *p* = 0.045). Predicted presence of *Anopheles* larvae is shown in Figure 4A.

### 3.2. Impact on Pupae

The pupal occupancy rates showed similar patterns as larval occupancy rates although occupancy rates differed among the three intervention arms at the start in round 1 (Control: 48.0%, CB: 34.9%, and ES: 10.6%). Nevertheless, these rates were virtually reduced to zero from round 5 of *Bti* application onwards in both CB (average 0.43%) and ES (average 0.27%) arms. At the same time, pupal occupancy rates remained high in the control from round 5 onwards with 11.0%, 10.6%, 12.5%, 0%, 16.7%, and 25.7% occupancy rates, respectively (Figure 3C,F,I).

### 3.3. Impact on Adult An. Gambiae

**Figure 5:**
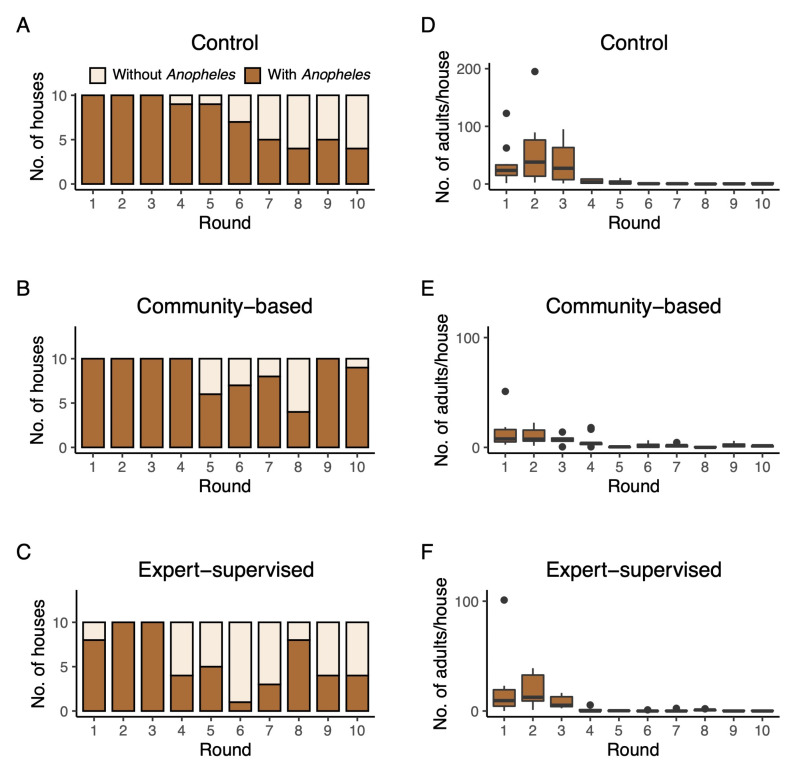
Number of houses with adult *An. gambiae* per survey round in the control arm (panel (**A**)) and number of adult *An. gambiae* per house per survey round in the control arm (panel (**D**)). Similarly, in panels (**B**,**C**,**E**,**F**), the same outcome parameters are shown for the CB arm and ES arm, respectively.

Besides the direct impact on larval and pupal populations, we also investigated a possible impact of *Bti* treatment on abundance of adult *An. gambiae* in houses neighboring the study arms. During the 10 rounds, on average, 73.0 ± 25.8% of control houses contained one or more *An. gambiae* over the two consecutive trapping nights. In CB and ES, these proportions were 84.0 ± 21.2% and 57.0 ± 30.9%, respectively (Figure 5A–C). Actual numbers of *An. gambiae* inside the houses were highly variable and could reach a two-night average of 195 individuals in round 2 in the control arm (Figure 5D). From round 5 onwards, numbers of adult *An. gambiae* reached near zero in all arms of the study (Figure 5D–F). A negative binomial model indicated that adult densities in control and ES arms declined equally fast over time (*p =* 0.468; Table 3). Densities in the CB arm declined significantly more slowly than in the control and ES arm. Predicted densities of adult *An. gambiae* per house are shown in Figure 4B.

## 4. Discussion

The current study explicitly aimed to assess the impact of *Bti* applied through an intervention program that was led and executed by the rice-farming community in comparison with an intervention program that was coordinated and supervised by an expert project team. The intervention trial with *Bti* covered one cycle of rice farming for a period of six months. *Bti* treatment was extended to other important mosquito breeding habitats of economic importance, such as water drains in inter-crop lands and hill water dams for rain water management. These were perceived by the local community as inadequate for habitat modification measures usually implemented by the local community [29].

During the study, a natural decline in larval populations over time was observed in the control, which can be explained by the fact that rice growth during the study inhibits oviposition on the available surface water by adult mosquitoes [38]. We therefore analyzed whether the reduction over time was stronger in the ES and CB arms than in the control arm of the study. The impact of *Bti* on *Anopheles* larvae was indeed significantly stronger in the expert-supervised arm of the study (estimated at 49% reduction per round) than in the community-based (28% reduction per round) and control arms (22% reduction per round). This suggests that careful implementation of larval source management by well-trained professionals had advantages over implementation by rice-farming communities. Apparently, the effectiveness of a larviciding program is reduced when implemented by communities themselves. This reduction may have numerous causes, including less careful application because of time constraints and other challenges related to incorporation into daily rice-farming practices.

In interviews carried out in parallel to the present study, several of these challenges were reported by *Bti* sprayer operators. Challenges were mainly related to the creation of pits for wetting vegetable crops at the bottom of the hills surrounding the rice fields as well as the new pools and puddles generated by rains. An upstream water dam constructed for storage of rain water and irrigation of the rice field was also not treated because this site was inaccessible to the operators with simple knapsack sprayers and required a motorized knapsack to ensure a long swath of the spray. The sprayers encountered specific challenges in rice habitats mainly related to the type of soil: in some areas or during a day with rain, the sprayer faced muddy and slippery soils. Walking along the narrow ridge of rice plots was reported as another obstacle, as it could limit full coverage of some larger rice plots. The interviews also confirmed a high increase in awareness and acceptance of mosquito control practices by the local community, especially because they were involved in the actual monitoring (larval dipping, etc.) and thus observed an impact. In addition, they perceived a high impact on mosquito nuisance and probably on malaria infection [27].

Results from pupal surveys support the larval survey data and showed that *Bti* application completely prevented development of larvae into pupae in both ES and CB arms, while in the control arm, substantial numbers of pupae were observed throughout the study. It should be noted that pupae themselves are not directly affected by *Bti* application, as they no longer take up food and are thus not ingesting the toxic crystal proteins that *B. thuringiensis* produces. The mass killing of larvae eventually inhibited pupal development and hence adult emergence from the treated sites. Interestingly, however, this reduction in adult emergence from breeding sites was not directly noticeable in houses that were sampled for adult mosquitoes and that were located nearby the rice fields. In fact, numbers of adult *An. gambiae* strongly declined in all arms of the study although less strongly in CB in comparison with ES and control. This overall population decline may be the result of weather changes during the six months of study as well as of all irrigated valleys in the area becoming less suitable for breeding of vectors. The relatively lower decline in CB may be explained by the fact that a small branch of the marshland near CB but located in an adjacent sector was not covered by the *Bti* intervention. As our study was a “non-randomized trial with control” with a relatively low number of sampled houses, the effect of *Bti* on adult populations could probably not be determined.

Mosquito population dynamics as observed in our study seem typical for populations from irrigated rice fields, e.g., the inner delta of the Niger River in Mali [38]. Here, the development of malaria vectors mostly took place in the first six weeks after transplantation of rice, then decreased as the plant height increased, and malaria vectors were almost absent close to the harvest of the rice. A re-establishment of *An. gambiae* s.l. occurred in small pools due to improper drainage during and after harvesting of the rice. A similar pattern was also observed in the Mwea rice scheme in Kenya by Mutero and colleagues, where the highest larval density was recorded in the three weeks post transplantation of rice seedlings, and then, the larval number dropped dramatically with development of rice until the harvesting period [39].

The impact of *Bti* on the aquatic stages of mosquitoes observed in the present study corresponds with results from studies conducted in urban and rural areas elsewhere in Africa [26]. In urban Dar es Salaam, Tanzania, larviciding resulted in a 96% reduction of anopheline larvae after one year of intervention although it had a moderate impact on malaria transmission [23]. The study highlighted that if the impact of larviciding is limited to *Anopheles* only, this may result in a lack of support from the community because the community does not perceive a direct, positive impact from a reduction in the intensity of human biting of *Culex* mosquitoes. In urban settings, the effect of larviciding should therefore be enhanced by environmental management measures and implemented by the community itself by controlling domestic mosquito breeding sites as well [40,41]. Besides the spatial extent of mosquito breeding sites being relevant to the success of *Bti*, timing of the application is also important. Larviciding conducted with *Bti* in the rural highlands of western Kenya contributed to a reduction of >90% in larval mosquito stages and of >80% in adult mosquitoes. The impact was less during the rainy season, and hence, the study suggested that larviciding may be more effective during the dry season and at the beginning of the rainy season for controlling malaria [24]. During this period, mosquito breeding sites are well-defined and contained [21]. In contrast, in flooded riverine habitats in the Gambia, the impact of larviciding on larval density was found to be moderate and without a major impact on malaria transmission. Application of the larvicide with simple spraying equipment was not effective in complex habitats that are unstable over time and difficult to access by foot [25].

## 5. Conclusions

*Bti* application by rice-farming communities themselves prevented emergence of malaria vectors from irrigated rice fields and other nearby habitats. Although the impact on the larval stages was less strong when the community-based study arm was compared with the expert-supervised arm, pupal production was completely prevented in both arms. Together with the willingness to financially contribute to the LSM program with *Bti* and the high perceived safety and acceptance of the product, our work presents evidence that, in an environment with limited resources, communities should become more engaged in malaria control initiatives. In Rwanda, rice farmers and leaders of community-based organizations are particularly suitable groups for implementing mosquito larval control activities. They should be empowered with minor resources in knowledge on vector ecology and larval source management. Larviciding for mosquito control can then be integrated into their farming activities and become equally effective as expert-based interventions.

## Figures and Tables

**Table 1:** Overview of the numbers of sampling points per transect per treatment.

Treatment	Location Name	Habitat Type	Transect	# Sampling Sites
Community-based 1	Gatare	Rice paddy	A	21
			B	22
			C	29
			Total	72
Community-based 2	Kizanye	Rice paddy	A	23
			B	23
			C	23
			Total	69
Expert-supervised 1	Kibaza	Rice paddy	A	51
			B	49
			C	48
			Total	148
Expert-supervised 2	Nyagafunzo	Water drains	A	5
			B	5
			C	5
			Total	15
Expert-supervised 3	Hill dams	Water dams		19
			Total	19
Control	Nyaburiba	Rice paddy	A	41
			B	41
			C	41
			Total	123

**Table 2:** Parameter estimates for the GLMM of occupancy rates of habitats with *Anopheles* larvae with sampling site included as random variable.

Variable (Control = Reference)	Parameter Estimate	Std. Error	*p*-Value
Intercept	0.882	0.881	0.317
Community-based	−0.163	1.083	0.880
Expert supervised	−0.412	1.035	0.690
Round	−0.248	0.025	<0.001
Round * Community-based	−0.074	0.037	0.045
Round * Expert-supervised	−0.424	0.050	<0.001

**Table 3:** Parameter estimates for the GLMM of adult *An. gambiae* per house with house included as random variable.

Variable (Control = Reference)	Parameter Estimate	Std. Error	*p*-Value
Intercept	4.383	0.291	<0.001
Community-based	−1.852	0.408	<0.001
Expert-supervised	−1.106	0.415	0.008
Round	−0.618	0.038	<0.001
Round * Community-based	0.321	0.049	<0.001
Round * Expert-supervised	−0.042	0.057	0.468

## Data Availability

The datasets used and analyzed in this manuscript are available from the Digital Archive Network Services (DANS-EASY) repository at https://doi.org/10.17026/dans-zta-mad4.

## Appendix A

**Table A1:** Table with main characteristics of the experimental sites, organizational structures of *Bti* application and entomology monitoring of larval and adult stages of mosquitoes in the study site. n.a., not applicable; CMAT, Community Malaria Action Team; ET, entomology technician.

	Control	Expert-Supervised	Community-Based
**Characteristics**			
Name of site (type of crop, size)	Nyaburiba (rice, 33 ha)	ES1—Kibaza (rice, 27 ha) ES2—Nyagafunzo (cropland, 8 ha) ES3—Water dams (n/a, 0.28 ha)	CB1—Gatare (rice, 25 ha) CB2—Kizanye (rice, 8 ha)
Total size	33 ha	35.3 ha	33 ha
***Bti* application**			
Organization of *Bti* sprayer teams	n.a.	4 teams of 3 sprayers 4 team leaders 2 porters 1 coordinator 1 project supervisor (not from community)	4 teams of 3 sprayers 4 team leaders 2 porters 2 coordinators
Logistic management	n.a.	Project research team	2 cooperatives of rice farmers
Reporting of *Bti* application	n.a.	Daily to research team	Weekly to research team
**Monitoring**			
Larval stages	3 members of CMATs 1 ET	6 members of CMATs 2 ET	6 members of CMATs 2 ET
Adult mosquitoes	2 members of CMATs 1 ET	2 members of CMATs 1 ET	2 members of CMATs 1 ET
